# A Single-Tube Nested PCR Method for *SMN1* Deletion Detection in Spinal Muscular Atrophy

**DOI:** 10.3390/mps9050128

**Published:** 2026-08-31

**Authors:** Ayano Kosaka, Makoto Sakima, Yoriko Noguchi, Tomoyoshi Shiroshita, Yasuhito Aoki, Yoshihiko Otsuka, Yoshihiro Bouike, Hisahide Nishio

**Affiliations:** 1 Faculty of Nutrition, Kobe Gakuin University, 518 Arise, Ikawadani-cho, Nishi-ku, Kobe 651-2180, Japan; aya915@nutr.kobegakuin.ac.jp (A.K.); n8yemx03@gmail.com (M.S.); 2 Department of Clinical Laboratory, Kobe University Hospital, 7-5-1 Kusunoki-cho, Chuo-ku, Kobe 650-0017, Japan; ynoguchi@med.kobe-u.ac.jp; 3 Sekisui Medical Co., Ltd., 2-1-3 Nihonbashi, Chuo-ku, Tokyo 103-0027, Japan; tomoyoshi.shiroshita@sekisui.com (T.S.); yasuhito.aoki@sekisui.com (Y.A.); Yoshihiko.Otsuka@sekisui.com (Y.O.); 4 Department of Occupational Therapy, Faculty of Rehabilitation, Kobe Gakuin University, 518 Arise, Ikawadani-cho, Nishi-ku, Kobe 651-2180, Japan; 5 Toyonaka AIWAEN Clinic, AIWAKAI Social Welfare Corporation, Toyonaka 561-0872, Japan

**Keywords:** spinal muscular atrophy, *SMN1* deletion, single-tube nested PCR

## Abstract

Spinal muscular atrophy (SMA) is a rare autosomal recessive neuromuscular disorder caused predominantly by homozygous deletion of *SMN1*, resulting in degeneration of lower motor neurons and progressive muscle weakness and atrophy. In recent years, newborn screening programs for SMA using dried blood spots and PCR-based assays have been introduced in several countries, enabling presymptomatic diagnosis and earlier initiation of therapy. However, newborn screening does not eliminate the need for diagnostic testing in routine clinical practice, because adolescents and adults with milder or ambulant phenotypes may still present only after symptom onset and may experience diagnostic delay. We therefore developed a single-tube nested PCR (STNPCR) method as a practical diagnostic approach for symptomatic patients with suspected SMA. This method enables detection of homozygous *SMN1* deletions using only standard PCR procedures and gel electrophoresis and may help identify patients with SMA who are not captured by newborn screening. Our assay using dried blood spot samples demonstrated preliminary technical feasibility in this proof-of-concept cohort, although further validation in larger independent cohorts will be required before its diagnostic utility can be established.

## 1. Introduction

Spinal muscular atrophy (SMA) comprises a group of genetic neuromuscular disorders. In this article, SMA refers specifically to 5q-associated SMA, a rare autosomal recessive neuromuscular disorder caused by biallelic alterations in the survival motor neuron 1 (*SMN1*) gene on chromosome 5q.

The incidence of 5q-SMA is approximately 1 in 10,000 live births, and its prevalence is estimated at 1 to 2 per 100,000 persons [1,2]. In approximately 95% of affected individuals, SMA is caused by homozygous loss of *SMN1*, most commonly due to deletion or gene conversion [3,4].

SMA was once considered an incurable disease; however, the introduction of disease-modifying therapies (DMTs) into clinical practice since 2016 has markedly changed its clinical management and highlighted the importance of presymptomatic diagnosis [5]. These therapies increase SMN protein levels, thereby ameliorating disease severity and improving motor outcomes and survival. Early initiation of treatment is critical in SMA [6]. Clinical studies in presymptomatic infants have shown that treatment initiated before symptom onset, including nusinersen and onasemnogene abeparvovec, is associated with better outcomes than treatment started after symptom onset [6,7,8]. In some infants, presymptomatic treatment was so effective that motor development approached the normal range. These findings strongly supported the introduction of newborn screening for SMA [9]. As a result, several countries have introduced newborn screening programs for SMA using dried blood spots and PCR-based assays.

However, newborn screening for SMA has not been implemented uniformly across countries and regions. Vrščaj et al. conducted a questionnaire survey of experts from 143 countries to assess the availability of DPTs and newborn screening for SMA in each country or region [10]. According to their report, 31 countries had implemented SMA newborn screening by 2023, and this number had increased to 33 by early 2024 [10]. These findings suggest increasing global recognition that newborn screening, together with early treatment, can improve clinical outcomes in SMA.

Nonetheless, implementation of newborn screening for SMA remains challenging in some countries because of limited governmental support, resource constraints, and differences in healthcare priorities. Therefore, a practical genetic testing strategy for symptomatic patients with suspected SMA remains necessary, particularly in regions without newborn screening programs and for individuals born before their implementation. Furthermore, even in countries where newborn screening has been introduced, older individuals with SMA who have already passed the neonatal period do not benefit from SMA newborn screening and may remain undiagnosed. Because they are no longer eligible for newborn screening, diagnosis may be delayed, limiting timely access to DMTs during the critical early stages of disease progression. All individuals with SMA should have the opportunity to benefit from DMTs. Regardless of age, patients with SMA-like symptoms should be evaluated to determine whether they may benefit from these therapies. Even if SMA is ultimately ruled out, such evaluation helps bring patients with SMA-like symptoms closer to an accurate diagnosis and appropriate medical care.

In such settings, establishing an accurate diagnosis is essential to expanding access to appropriate medical care. Therefore, we developed a practical genetic testing strategy for symptomatic patients with suspected SMA, in which STNPCR is performed directly from a filter-paper blood spot in a single tube. In this study, we focused exclusively on a combined system of conventional PCR and agarose gel electrophoresis. Conventional PCR with gel electrophoresis is suitable for low-throughput diagnostic testing. The results clearly demonstrated the feasibility of this approach and supported its use as a practical diagnostic method for patients with SMA-like symptoms.

## 2. Materials and Methods

### 2.1. Residual DBS Samples and Ethics Committee Approval

A total of 20 residual dried blood spot (DBS) samples were used in this study. The samples included DBS samples from 10 SMA patients and 10 controls. Patients were diagnosed with *SMN1*-deficient SMA based on *SMN1* deletion testing using fresh blood by PCR-restriction fragment-length polymorphism (PCR-RFLP) analysis [11]. Controls are non-SMA individuals confirmed by PCR-RFLP, which means individuals carrying at least one copy of *SMN1*.

Although the samples had been anonymized, this study was made public through an opt-out procedure. This study was approved by the Ethics Committee of Kobe University Graduate School of Medicine (reference B230027, approved on 14 June 2023) and the Ethics Committee of Kobe Gakuin University (regarding this study conducted with residual DBS samples in Kobe Gakuin University: reference 23-02, approved on 16 October 2023). The present study was conducted in accordance with the World Medical Association Declaration of Helsinki.

### 2.2. Outline of the STNPCR System

**Figure 1:**
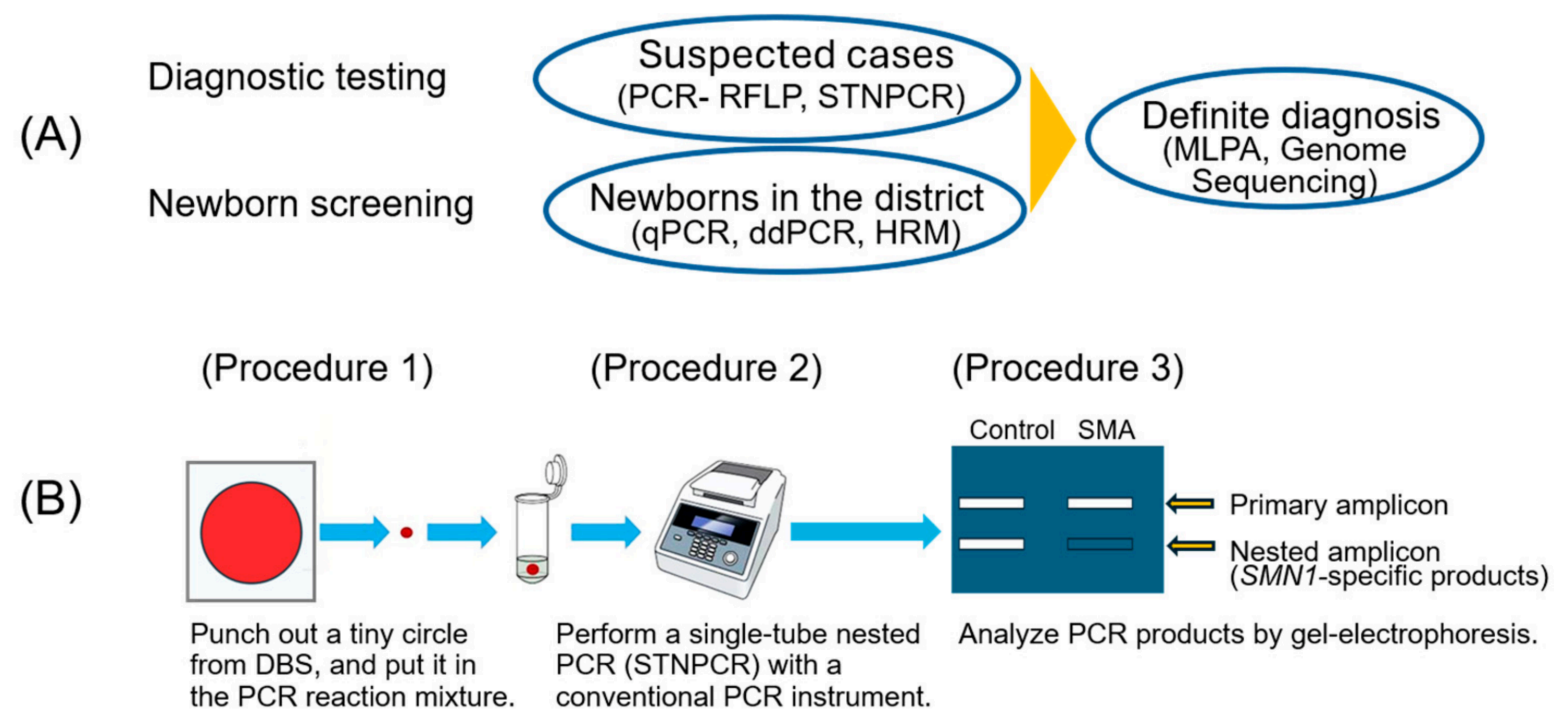
Positioning and workflow of single-tube nested PCR (STNPCR) for SMA diagnosis. (**A**) Position of the STNPCR system among diagnostic methods for SMA. (**B**) Outline of the diagnostic workflow for SMA using STNPCR. Other abbreviations than STNPCR are as follows: PCR-RFLP: PCR-restriction fragment-length polymorphism, qPCR: quantitative PCR, ddPCR: droplet digital PCR, HRM: high-resolution melting analysis, MLPA: Multiplex Ligation-dependent Probe Amplification.

The STNPCR system developed in this study may be useful as an initial test for patients suspected of having SMA in the diagnostic reasoning process. Figure 1A shows the position of STNPCR in comparison to other SMA diagnostic methods.

The STNPCR system consists of three procedures (Figure 1B): (1) preparation of a tiny DBS circle and placing it directly into a PCR reaction mixture; (2) performing STNPCR in a conventional PCR or real-time PCR instrument; and (3) detecting the presence or absence of the *SMN1* gene by gel electrophoresis of STNPCR products.

**Figure 2:**
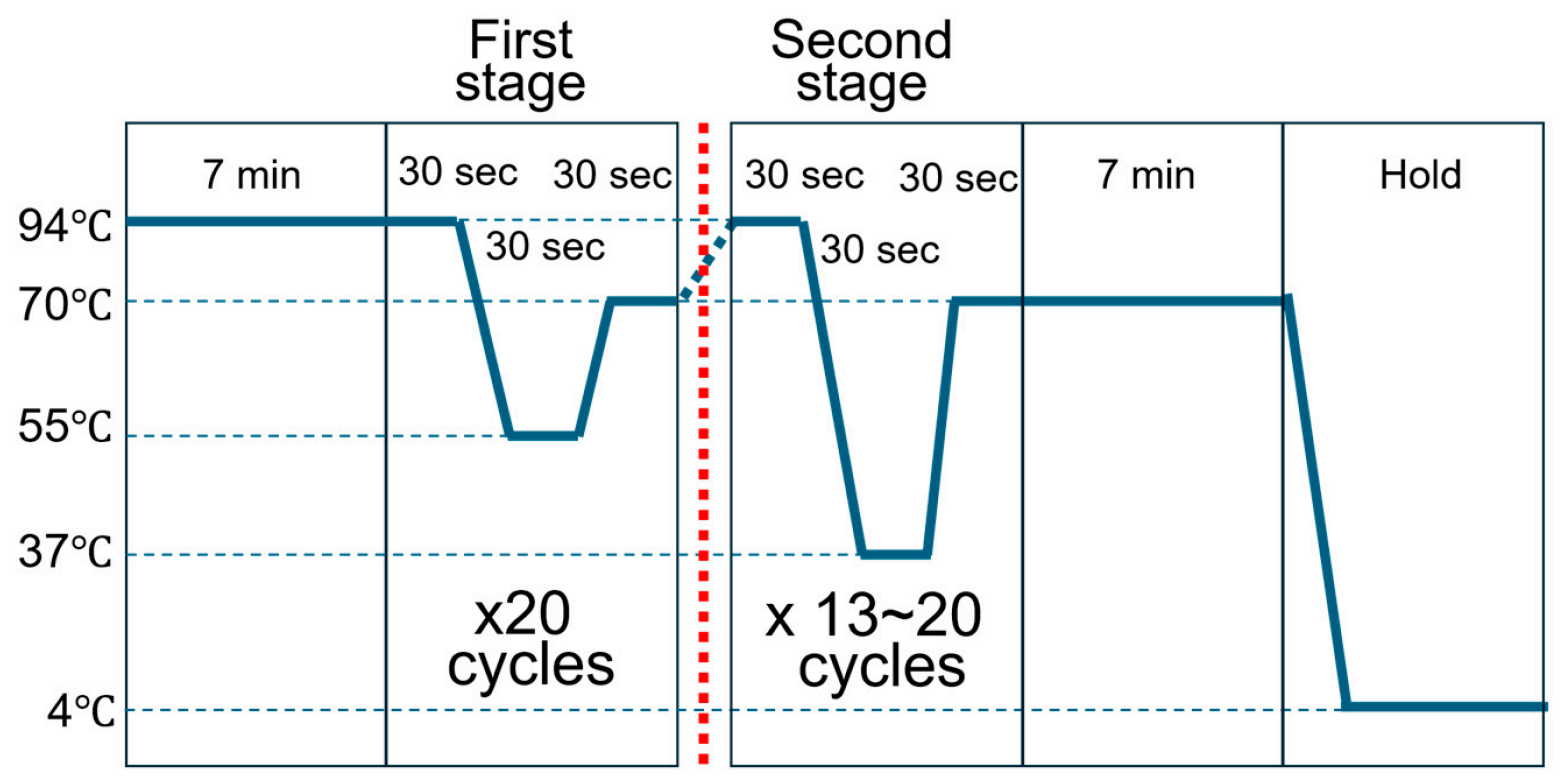
Schematic representation of the PCR cycling conditions of the two-stage STNPCR. The program included an initial denaturation step, a first amplification stage at 55 °C, a second amplification stage at 37 °C, an additional extension step, and a final hold at 4 °C. The red dotted line indicates the timing when the first-stage PCR ends and the second-stage PCR begins.

**Figure 3:**
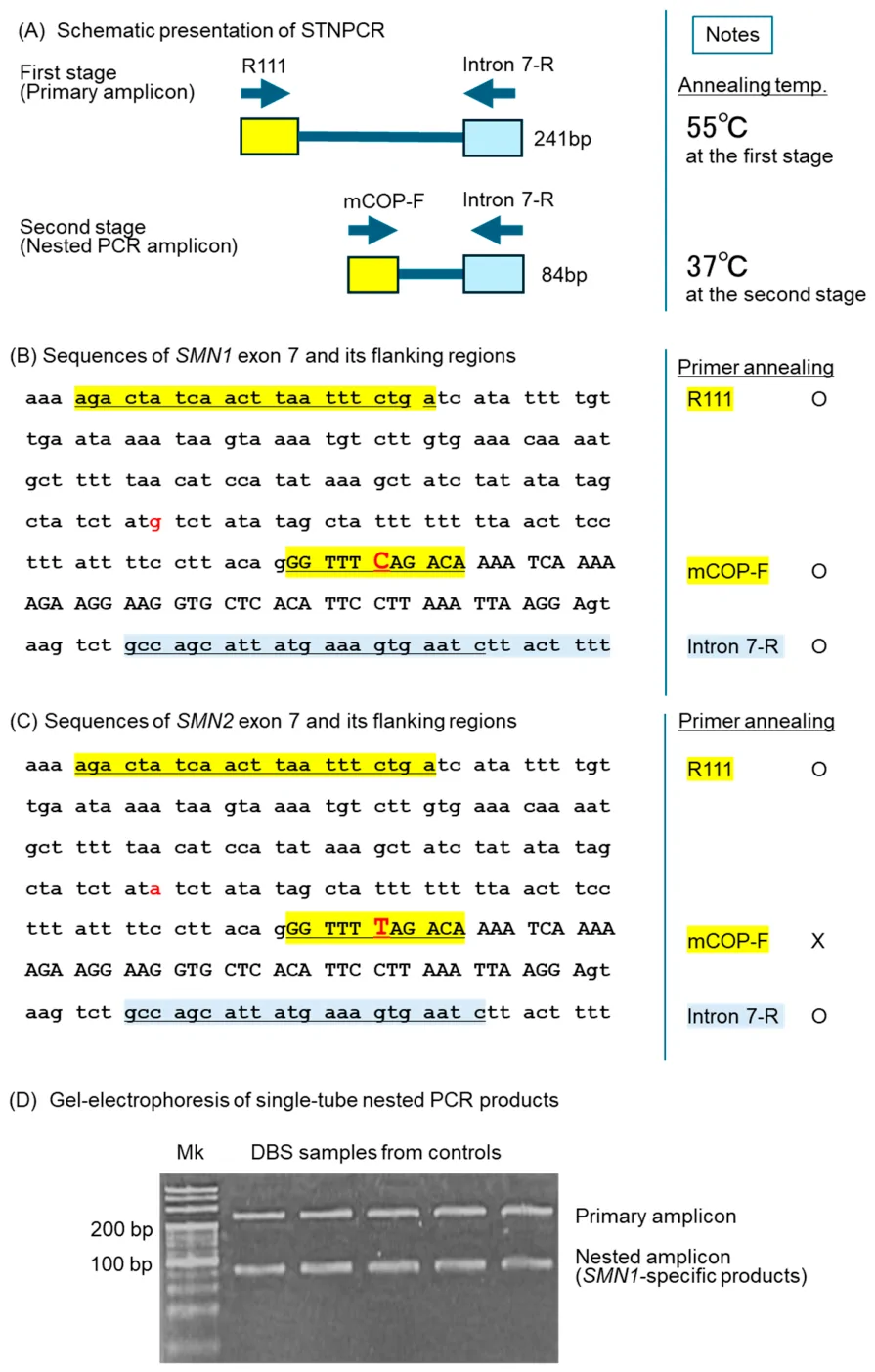
STNPCR assay using DBS samples from control individuals. (**A**) Schematic diagram of STNPCR. (**B**,**C**) Alignment of the corresponding *SMN1* and *SMN2* target regions analyzed in this study. Uppercase letters indicate exon sequence, whereas lowercase letters indicate flanking intronic sequence. The sequences were annotated with reference to Ensembl gene models on the human genome assembly GRCh38. *SMN1* and *SMN2* correspond to Ensembl gene IDs ENSG00000172062 and ENSG00000205571, respectively. The single-nucleotide difference within the exonic region corresponds to the known *SMN1/SMN2* sequence distinction at c.840. Sequences highlighted in yellow are forward primers (R111 and mCOP-F), and sequences highlighted in blue are reverse primers (intron 7-R). Red letters represent different nucleotides in the *SMN1* and *SMN2* genes. The mCOP-F primer is an *SMN1*-specific primer, annealing with C nucleotide at c.840 in *SMN1* exon 7, but not with T nucleotide at c.840in *SMN2* exon 7. The underlines indicate the positions of the primers. (**D**) Gel electrophoresis data of STNPCR products. DBS samples were collected from control subjects. In this experiment, an STNPCR were performed with 20 cycles for the first stage and 20 cycles for the second stage. Mk indicates a molecular marker.

STNPCR includes two amplification stages (Figure 2 and Figure 3A). At the first stage with an outer primer set, a longer sequence containing a mixture of *SMN1* and/or *SMN2* sequences is amplified by asymmetric PCR. This sequence is always amplified because no individuals lack *SMN1* and *SMN2*. Thus, in *SMN1*-deleted SMA patients, the primary amplicon is derived from *SMN2*. In the second stage with an inner primer set, a shorter sequence including an *SMN1*-specific nucleotide is amplified (nested amplicon). The forward primer is a short *SMN1*-specific primer (modified competitive oligonucleotide priming primer, mCOP-F in Section 2.4), which binds to *SMN1* specifically. To check the STNPCR results, gel electrophoresis was used.

### 2.3. Preparation of a Punched Circle from DBS

Approximately 100 μL of blood was collected from controls and SMA patients, and spotted onto filter paper (FTA^®^ Elute Cards; GE Healthcare, Boston, MA, USA). This filter paper can store DBS for more than 10 years. Prior to this study, we conducted basic experiments related to SMA screening using DBS on this type of filter paper [11].

### 2.4. Instrument and Primers for the PCR Experiments

PCR was performed using the Mastercycler^®^ Nexus (Eppendorf SE, Tokyo, Japan). The first stage primers were used to amplify the target sequence containing *SMN1*/*SMN2* exon 7: R111 (5′-AGA CTA TCA ACT TAA TTT CTG ATC A-3′) and Intron 7-R (5′-GAT TCA CTT TCA TAA TGC TGG-3′) [2,12]. The second stage primers were used to amplify the sequence containing an *SMN1*-specific nucleotide: mCOP-F (5′-GGT TTC AGA CA-3′) and Intron 7-R.

### 2.5. PCR Reaction Mixture

A punched circle (1.2 mm in diameter) from a DBS on filter paper was directly placed into the PCR reaction mixture containing 1 U DNA polymerase KOD FX Neo™ (Toyobo, Osaka, Japan) to a final volume of 25 μL (Table 1). Three primers (R111, Intron 7-R, mCOP-F) were also included in the PCR reaction mixture. The full reaction compositions are shown in Table 1.

### 2.6. PCR Conditions

PCR was performed in a 25 µL reaction mixture under the following conditions: (1) initial denaturation at 94 °C for 7 min; (2) the first stage: 20 cycles of denaturation at 94 °C for 30 s, annealing at 55 °C for 30 s, and extension at 70 °C for 30 s; (3) the second stage amplification: 13−20 cycles of denaturation at 94 °C for 30 s, annealing at 37 °C for 30 s, and extension at 70 °C for 30 s; (4) additional extension at 70 °C for 7 min; and (5) hold at 4 °C.

**Figure 4:**
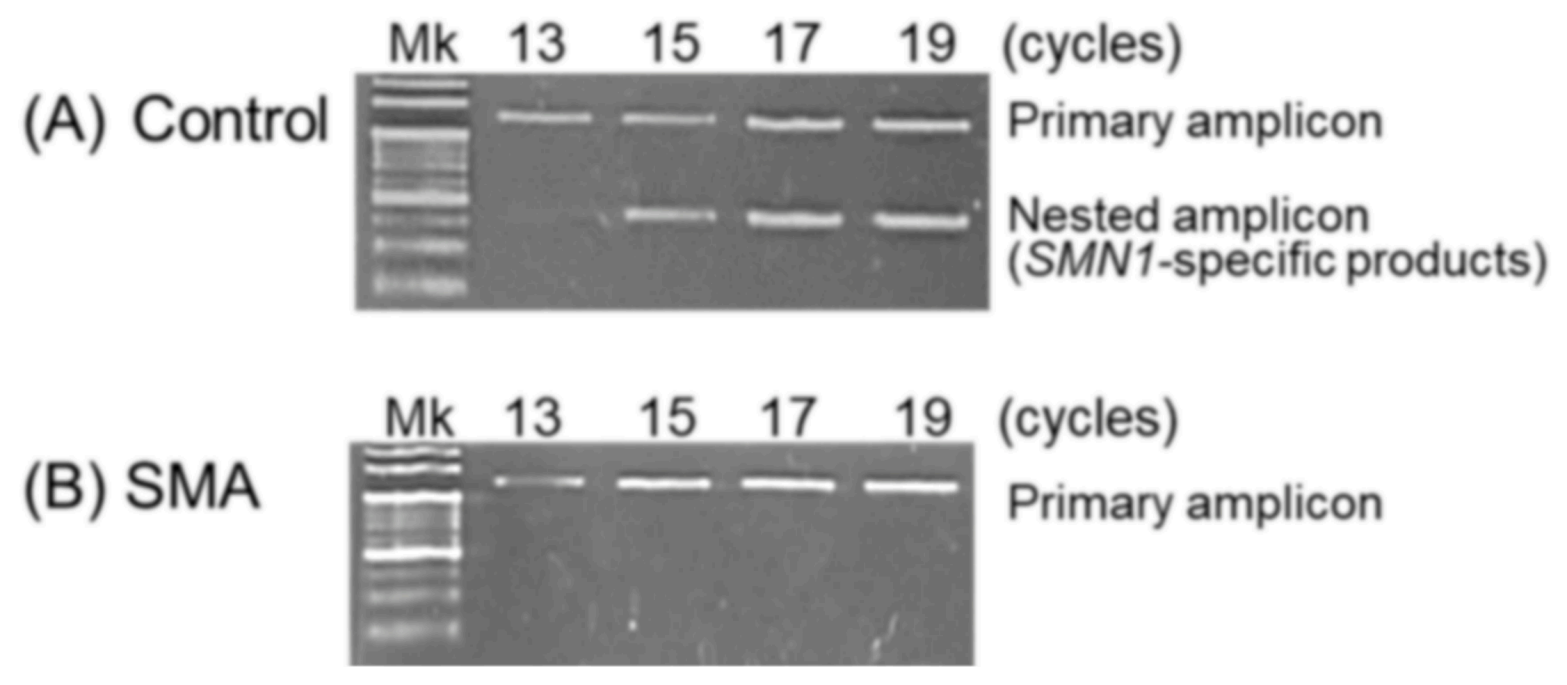
Cycle numbers of the second stage of DBS samples. (**A**) Control individuals. (**B**) SMA patients. Second-stage cycles of 15–19 showed two bands of the primary amplicon and nested amplicon (*SMN1*-specific products) in the control DBS sample, but a single band of the primary amplicon in the SMA DBS sample. However, 13 cycles of the second stage in the control DBS showed a very faint band (almost undetectable) of nested amplicon (*SMN1*-specific product band), which may lead to a false-positive result. Mk denotes molecular markers.

In our STNPCR system, asymmetric PCR was employed in the first stage using the outer primer pair R111 (30 nM) and Intron 7-R (300 nM), whereas conventional PCR was performed in the second stage using the inner primer mCOP-F (300 nM) and Intron 7-R (300 nM). The asymmetric conditions in the first stage were designed to minimize residual R111 primer and thereby prevent interference with mCOP-F annealing during the second-stage reaction. The 241 bp amplicon generated by the outer primer pair in the first stage was also available as a template for the second-stage reaction. During the second stage, the nested 84 bp *SMN1*-specific amplicon was generated. Notably, the 241 bp outer amplicon could also be re-amplified during the second stage, and its intensity increased with increasing numbers of second-stage cycles (Figure 4).

### 2.7. Gel Electrophoresis

The final PCR product was then subjected to gel electrophoresis together with a molecular marker (20 bp DNA Ladder; Takara Bio Inc., Shiga, Japan) and visualized using FAS-Digi PRO (NIPPON Genetics Co. Ltd., Tokyo, Japan) after staining with Midori-Green Dye (NIPPON Genetics Co. Ltd.). At least “one no template DNA” control was included in the experiments and showed no amplification, even though it was not displayed in the figures.

## 3. Results

### 3.1. Trial Experiments with STNPCR Technology

STNPCR assay results using DBS samples from control individuals are shown in Figure 3. Figure 3A shows a schematic diagram of STNPCR system. The first-stage amplification generated a 241 bp primary amplicon, and the second stage amplified an 84 bp nested amplicon (*SMN1*-specific product).

Figure 3B,C show the sequences of *SMN1* and *SMN2* exon 7 and their flanking regions. Sequence comparison reveals two nucleotide differences: one located in intron 6 and the other in exon 7. Specifically, *SMN1* contains G in intron 6 and C in exon 7, whereas *SMN2* contains A in intron 6 and T in exon 7. These differences are highlighted in red. The mCOP-F primer specifically anneals to the C nucleotide in exon 7 of *SMN1*.

Figure 3D shows the gel electrophoresis results for the trial experiments using control samples. In this case, STNPCR were performed with 20 cycles for the first stage and 20 cycles for the second stage. We successfully obtained two bands of primary amplicon and nested amplicon (*SMN1*-specific products).

### 3.2. Optimization of the Cycle Number for the Second-Stage Amplification

To avoid false-positive and false-negative results, we must determine the optimal cycle number of the first stage and second stage. In this case, we fixed the cycle number for the first stage amplification to 20 cycles and changed the cycle number of the second stage from 13 to 19 cycles.

On the gel electrophoresis, 15–19 cycles of the second stage showed two distinct bands of the primary amplicon and nested amplicon (*SMN1*-specific products) in the control DBS sample and a single band of the primary amplicon in the SMA DBS sample (Figure 4). However, 13 cycles of the second stage in the control DBS sample showed a very faint band (almost undetectable) of nested amplicon (*SMN1*-specific products), which may lead to a false-positive result. In this experiment, no bands due to nonspecific amplification products were observed. On the basis of these results, subsequent experiments were performed using 15 cycles for the second-stage amplification.

### 3.3. Establishment of P/A Assay of SMN1 Using DBS Samples

On the gel electrophoresis of STNPCR products, the controls show two bands, outer-primer-set products of 241 bp (primary amplicon) and inner-primer-set products of 84 bp (nested amplicon, or *SMN1*-specific products) (Figure 3 and Figure 4), while SMA patients show only one band, an outer-primer-set product of 241 bp. No inner-primer-set product of 84 bp was observed in SMA patients.

**Figure 5:**
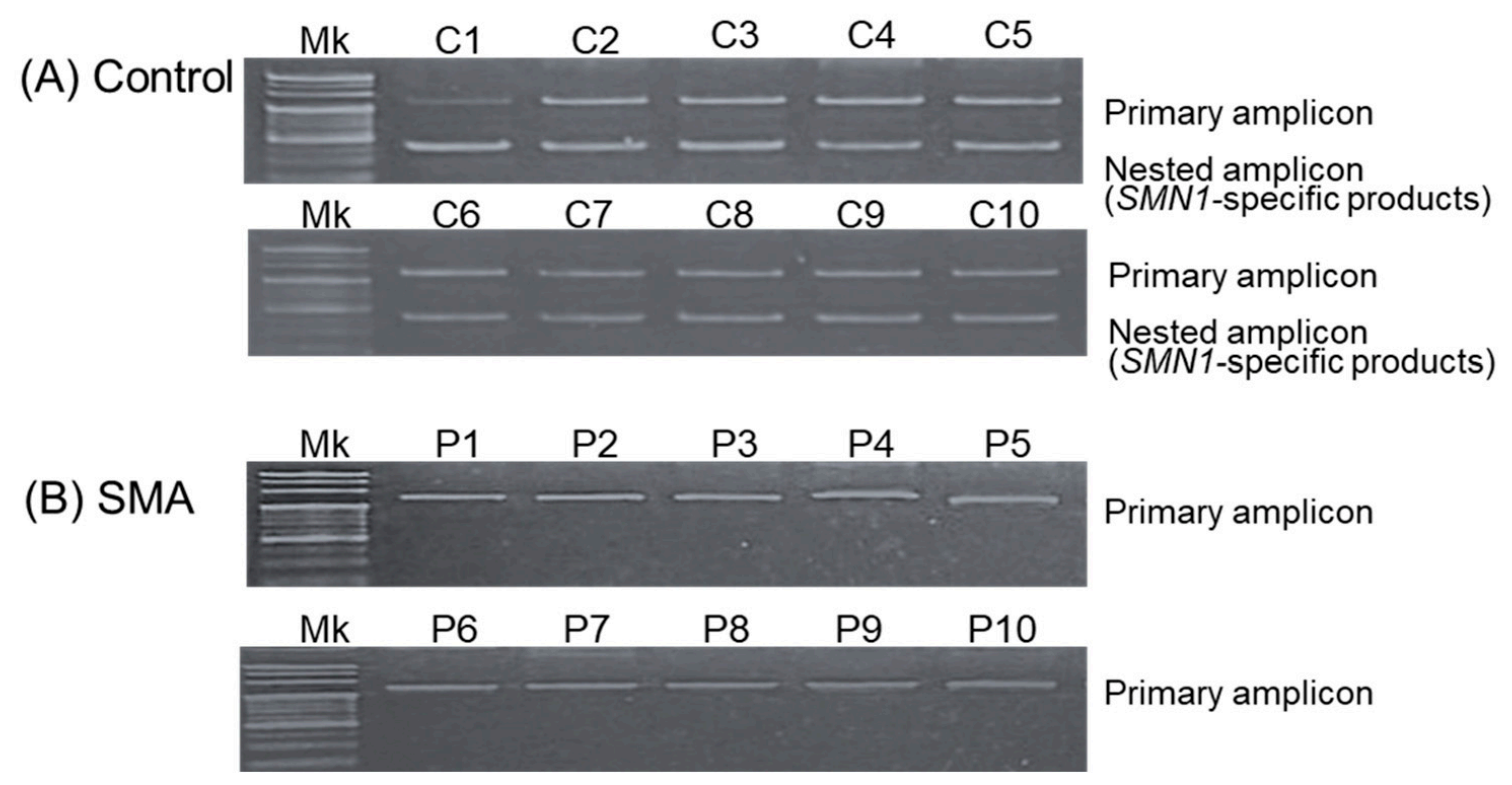
P/A assay of *SMN1* using conventional PCR. (**A**) Control individuals. (**B**) SMA patients. The results were as expected based on RFLP analysis. However, a clear upper band was not observed in some DBS samples (C1). Although *SMN1*-specific products were clearly shown in the C1 sample, this sample needs to be re-examined to test the possibility of false results. The C1 sample was confirmed to be from control samples. The variation in the band intensity likely reflects differences in total *SMN* gene copy number or template quantity in the DBS sample.

Figure 5 shows the results of the presence and absence (P/A) assay of *SMN1* using DBS samples collected from the controls and SMA patients. The experiments were performed with 20 cycles for the first stage and 15 cycles for the second stage. All DBS samples from SMA patients showed only a band of primary amplicon, with no band of nested amplicon (*SMN1*-specific products).

Some variability in band intensity can occur when using DBS as a template and that such variability does not necessarily indicate *SMN1* deletion. Careful interpretation of the results is essential to avoid false conclusions. In particular, atypical band patterns should be carefully examined, as they may indicate underlying issues. In this context, the relative intensity of the two bands in control samples is an important factor. As shown in Figure 3 and Figure 4, most control samples exhibited two distinct bands. However, in the “13-cycle” condition (Figure 4) and sample C1 (Figure 5), one of the bands was extremely weak (almost undetectable), indicating insufficient signal strength to reliably confirm a non-SMA condition.

## 4. Discussion

### 4.1. Positioning of This Study

Our original concept, first reported in 2019 [12], was based on two core principles. First, when only a limited amount of DNA is available, two-tube nested PCR enables reliable amplification of the target sequence. Second, the short mCOP primer has high sequence specificity and is unlikely to anneal to templates containing even a single-nucleotide mismatch. In our initial study, DNA was first pre-amplified in one tube, and the target sequence was then specifically amplified in a separate tube using a short mCOP primer. Thus, the P/A assay required two separate amplification steps.

The STNPCR system described in this study integrates the two amplification rounds of the previous two-tube nested PCR system. Its first stage corresponds to the first round, and its second stage corresponds to the second round. Importantly, the entire process is completed within a single tube. Although converting nested PCR from a two-tube format to a single-tube format may appear to be a minor technical modification, it has important practical implications.

### 4.2. Single-Tube Nested PCR (STNPCR) with a Punched Circle from DBS

This single-tube approach is made possible by the use of an mCOP primer. The mCOP primer used in our systems is approximately 10 nucleotides long, intentionally shorter than standard PCR primers of 20 to 30 nucleotides, with the gene-specific nucleotide positioned at its center [13,14]. The use of such short primers enables selective amplification of sequences containing the gene-specific nucleotide. As a result, these primers are less likely to anneal efficiently to templates containing even a single-nucleotide mismatch. In addition, the mCOP primer functions at an annealing temperature of around 37 °C, which is substantially lower than that used in the first-round amplification. By exploiting this difference in annealing temperature, STNPCR can be performed within a single tube.

Our STNPCR system requires only that a punched circle from a DBS be placed into the reaction tube, after which PCR is performed without DNA extraction to specifically amplify the target *SMN1* sequence. The use of DBS made it possible to perform the P/A assay even when only a limited amount of DNA was available. In addition, keeping the tube closed throughout the procedure minimizes the risk of contamination [15].

To improve assay reliability, the cycle conditions were carefully optimized to reduce the risk of false-positive and false-negative results. These technical precautions support the practical use of this system, although further evaluation in a larger number of clinical samples will be necessary to fully assess its diagnostic performance.

### 4.3. Limitations

Despite the practical advantages of this system, several limitations of the present study should be acknowledged.

Our P/A assay for *SMN1* detects only the presence or absence of *SMN1* and cannot determine the copy numbers of *SMN1* or *SMN2*. Therefore, it cannot identify SMA carriers or predict disease severity, which is influenced by *SMN2* copy number. Nevertheless, the assay may still be useful as an initial screening tool for the rapid identification of individuals lacking *SMN1*.

Because this study focused on method development and proof of concept, a small number of DBS samples from control individuals and SMA patients was sufficient to demonstrate the feasibility of the *SMN1* P/A assay. However, the current cohort size was insufficient for a definitive evaluation of false-positive and false-negative rates, diagnostic sensitivity and specificity, or robustness against the full range of DBS-associated variability and inhibitory factors. Further validation in a larger clinical cohort will be necessary to establish its diagnostic performance in routine practice.

## 5. Conclusions

In this study, we developed a DBS-based STNPCR system for the detection of homozygous *SMN1* deletion without DNA extraction. This system enables simple single-tube analysis using a punched circle from a DBS and may reduce the risk of contamination while simplifying the workflow in routine hospital laboratories. Although the STNPCR system has not yet been fully established as a diagnostic assay, the present findings suggest that the DBS-based STNPCR system may provide a simple, inexpensive, and practically applicable approach for the evaluation of patients with suspected SMA in routine hospital laboratories.

## Figures and Tables

**Table 1:** Reaction mixture for STNPCR.

Components	Volume (μL)
Distilled water	4.25
2 × PCR buffer for KOD FX Neo (Toyobo)	12.50
KOD FX Neo (1.0 U/μL) (Toyobo)	0.50
2 mM dNTPs (Toyobo)	5.00
R111 (1.0 pmol/μL)	0.75
Intron 7-R (10.0 pmol/μL)	0.75
mCOP-F (10.0 pmol/μL)	0.75
DBS: 1 piece (1.2 mm diameter)	0.50
Total	25.00

DBS, Dried blood spot; mCOP, modified competitive oligonucleotide priming.

## Data Availability

The data that support the findings of this study are available from the corresponding author, H.N., upon reasonable request.

## Abbreviations

The following abbreviations are used in this manuscript:

SMA	Spinal muscular atrophy
DMT	Disease-modifying therapy
NBS	Newborn screening
STNPCR	Single-tube nested PCR
DBS	Dried blood spot
PCR-RFLP	PCR-restriction fragment-length polymorphism
qPCR	Quantitative PCR
ddPCR	Droplet digital PCR
MLPA	Multiplex ligation-dependent probe amplification
P/A	Presence and absence
mCOP	Modified competitive oligonucleotide priming
